# Combating climate-induced health threats through Co-Constitutive Risk (CCR) Messaging: A *One Health Communication* approach

**DOI:** 10.1371/journal.pntd.0012676

**Published:** 2024-12-02

**Authors:** Asheley R. Landrum, Dominik A. Stecuła, Matthew Motta

**Affiliations:** 1 Walter Cronkite School of Journalism & Mass Communication, Arizona State University, Phoenix, Arizona, United States of America; 2 School of Communication, Ohio State University, Columbus, Ohio, United States of America; 3 Department of Health Law, Policy, & Management, Boston University School of Public Health, Boston, Massachusetts, United States of America; University of California Berkeley, UNITED STATES OF AMERICA

## Abstract

Climate-facilitated spread of mosquito-borne pathogens to new environments elevates the importance of policies that limit greenhouse gas emissions as well as the development and uptake of new pharmaceutical interventions. Whereas past research attempts to bolster support for both through either climate or health risk communication, fewer attempt to *combine* the risks borne by climate change *and* infectious disease with a single messaging intervention, i.e., co-constitutive risk messaging (CCR), a strategy of an integrative approach we call *One Health Communication*. In a pre-registered experiment embedded in a nationally representative survey (N = 2,200), we test whether CCR messaging impacts support for pharmaceutical interventions to minimize dengue fever health risks and/or policy efforts to mitigate climate change. We find that CCR messages are generally effective at increasing support for pharmaceutical interventions aimed at ameliorating the health risks posed by dengue fever. Moreover, we find suggestive evidence that people who hold more collectivistic worldviews are especially receptive to messages that emphasize the public (vs. the personal) health risks of dengue fever. In *post hoc* exploratory analyses, we show that CCR messages affect public support for climate change mitigation policies among those who express doubts about human-caused climate change. We conclude by discussing the usefulness of CCR messaging and One Health Communication approaches more broadly in various strategic communication contexts and considering additional avenues for future research.

## Author summary

Our research explores the potential of co-constitutive risk messaging (CCR), a method within our One Health Communication framework, to influence public support for both immediate actions like vaccine uptake and broader policies such as climate change mitigation. While we observed that CCR messaging can effectively enhance pro-vaccine attitudes and behaviors, especially among those with collectivist cultural views, its impact on climate policy support was more nuanced. Notably, CCR messaging appears to resonate with individuals who are skeptical about climate change, suggesting that linking climate change to specific health risks like infectious diseases may make the abstract consequences of climate change more tangible and urgent for this group. Although our findings provide mixed support for our initial hypotheses, they represent a promising first step in evaluating CCR messaging as a strategy to address complex, interlinked issues like climate change and public health. We advocate for further research to refine and expand the application of CCR messaging specifically, and One Health Communication more broadly, potentially incorporating stronger experimental treatments and exploring its effectiveness across different cultural contexts. This approach could offer a valuable tool for strategic communicators aiming to engage diverse audiences on the intertwined challenges of health and environmental risks.

## Introduction

*One Health*, as defined by the Centers for Disease Control and Prevention (CDC) [1] and the World Health Organization (WHO) [2], embodies an integrated approach that recognizes the interdependence of human, animal, and environmental health. Interdisciplinary and transdisciplinary efforts have employed this approach to study issues such as antimicrobial resistance, vector-borne diseases, food animal diseases, and water contamination [1,2]. Limited research, however, has explored how the intricacies of One Health issues, specifically, can be effectively communicated to diverse audiences. To address this gap, we propose *One Health Communication* as a new approach to enhancing traditional health communication in these contexts.

### One Health and Co-Constitutive Risks

According to the CDC [3], the prevalence of vector-borne diseases is increasing due to rising temperatures facilitating the expansion of mosquito and tick habitats. Global climate change facilitates the spread of mosquito-borne pathogens that cause several forms of hemorrhagic fever—including dengue fever—to new environments [4], including parts of the United States. For instance, the CDC documented several isolated outbreaks of dengue fever in the US over the past several years, in the continental US (e.g., Texas, 2013) and Hawaii (in 2015) [5]. The increased prevalence in the US of what researchers call “neglected tropical diseases”, like dengue fever, could represent a major public health concern [6]. Dengue, specifically, is concerning not only due to the disease’s high mortality rate, as virtually all those reinfected with the virus ultimately succumb to the illness [7], but because there is no known cure for the disease [8]. Combating the climate change-induced public health challenges of diseases like dengue, therefore, entails preventing disease onset by pursuing policies that either (a) rely on pre-existing vaccines to form the basis of mass childhood vaccination campaigns and/or (b) make an effort to mitigate rising global temperatures attributable to climate change.

### Communicating risks to increase policy support

Although a frequent goal of health communication campaigns is to promote individual health-related behaviors (e.g., smoking cessation), such campaigns also can aim to increase support for specific policies that, if enacted, intervene at a societal level (e.g., ending the sale of flavored tobacco products, requiring labels on tobacco products). One way that typical health communication messages achieve these goals is by communicating potential harms and/or the relative risk of those harms [9]. Traditional messages aiming to mitigate the spread of dengue may choose to emphasize the relative risks of contracting it. A One Health Communication approach, however, emphasizes the interconnected, or co-constitutive, nature of the risks inherent in One Health issues. For example, such an approach could highlight both the immediate risks of contracting dengue as well as the broader impact of climate change on those risks and, thus, aim to encourage support for policies targeting both mitigation of the disease and of climate change.

A variety of theoretical frameworks have been used to design messages and examine their effectiveness in health-related contexts. Although messages and campaigns focused on changing health behaviors are often informed by the Health Belief Model [10–12] or the Theory of Planned Behavior [13,14], those focused on increasing or measuring policy support are often informed by (versions of) Framing Theory [15,16] and/or differential receptiveness theories [17] such as identity-protective reasoning [18,19] and the cultural cognition theory of risk [20], particularly as it is generally accepted that policy support is heavily influenced by cultural worldviews and political ideology [21,22].

In this study, we examine the potential effectiveness of a One Health Communication approach, co-constitutive risk messaging in the context of dengue fever, to influence support for vaccination policies to mitigate the potential proximal harms of dengue and green energy policies to mitigate the broader impacts of climate change. Because our outcome of interest is policy support, and not specific health behaviors, our study is informed by the cultural cognition theory of risk and message framing.

#### Cultural cognition theory of risk and message framing

The *Cultural Cognition Theory of Risk* [20,23] posits that individuals’ perceptions of risks and benefits associated with various policies, technologies, and societal choices are shaped by their cultural values and group identities. That is, people are motivated to adopt beliefs about societal risks that resonate with the views and interests of groups with which they identify, reinforcing their connections within these groups. Though there are many ways in which values and worldviews could be ordered or grouped, this theory typically categorizes cultural values along two dimensions: from egalitarian to hierarchist and from individualist to communitarian. Greater egalitarianism reflects prioritization of equality, whereas greater hierarchism reflects preference for a structured distribution of roles; greater individualism reflects emphasis on personal freedoms and responsibility, whereas greater communitarianism prioritizes collective responsibility and well-being [20]. In the current study, we focus only on the individualist to communitarian dimension.

Understanding cultural orientations can help in crafting effective messages by aligning the framing of risks and interventions with targeted audiences’ worldviews. Again, cultural worldviews significantly influence how individuals perceive risks and assess the acceptability of interventions aimed at mitigating these risks [24–26], consistent with motivated reasoning [27]. People with a strong individualist orientation may respond more positively to messages that frame risks in terms of personal consequences rather than collective dangers. Such individuals also may be more likely to resist interventions that they perceive as infringing on their personal freedoms, such as mandatory masking or bans on plastic straws. On the other hand, those with communitarian inclinations may be more receptive to framing that emphasizes the collective impact of risks and may show greater willingness to accept restrictions on their personal freedoms if these are seen as benefiting the wider community.

A key challenge with co-constitutive risk messaging, however, is the potential for boomerang effects [28], where messages that bundle different risks can lead different audiences to perceive a lower risk than intended [26]. For example, experimental studies examining media frames of the Zika virus [26] showed that when the risk was linked to global climate change, participants who scored higher on hierarchism and individualism (i.e., hierarchist-individualists) tended to downplay Zika’s dangers compared to when they were presented with information solely about its public health risks. However, these same individuals showed increased concern when Zika was framed as being exacerbated by immigration. Conversely, those participants who scored lower on hierarchism and individualism (and thus, higher on egalitarianism and communitarianism, i.e., egalitarian-communitarians) demonstrated consistent levels of concern for Zika, whether it was framed solely as a public health issue or linked to global climate change. Associating Zika risks with increased immigration, however, led egalitarian-communitarians to underestimate its threat compared to the public health framing alone. Thus, how risks are framed and/or connected can significantly influence public perception, particularly when these connections resonate differently across cultural groups.

### Current study

The messages in this study were designed to emphasize the risk faced by either the individual or the general US adult population (collective) and suggest potential actions (policies) that might help mitigate the threat.

The first set of hypotheses predict that co-constitutive risk (CCR) messaging, the One Health Communication strategy we test in this study, will be effective for increasing policy support for the immediate risk of dengue fever (e.g., vaccine policies) and for addressing the superordinate conditions created by climate change that exacerbate the threat (climate policies).

*Hypothesis 1.* ***Immediate Outcomes*: *Effects on Support for Vaccination Policies*.** Exposure to CCR messages that emphasize either the personal health risks or collective health risks of mosquito-borne illness attributable to climate change will be associated with increased (a) support for investment in dengue vaccine research, (b) intention to vaccinate against dengue, and (c) support for expanding dengue vaccination mandates.
*Hypothesis 2.* ***Superordinate Outcomes*: *Effects on Support for Climate Change Mitigation Policies*.** Exposure to CCR messages that emphasize either the personal health risks or collective health risks of mosquito-borne illness attributable to climate change will be associated with increased support for climate change mitigation policy.

Furthermore, the expected asymmetry in how Individualists versus Communitarians would respond to personal versus collective framing of risk [23] is reflected in Hypothesis 3.

*Hypothesis 3.* ***Moderation by Cultural Cognitive Orientations*.** The effects of CCR messaging should be moderated by cultural cognitive orientations, such that Individualists are more likely to exhibit the aforementioned effects on (a) immediate and (b) superordinate outcomes when exposed to CCR messages that emphasize personal risk, while Communitarians should be more likely to exhibit effects on support for (c) immediate and (d) superordinate interventions when exposed to CCR messages that emphasize collective risk.

## Methods

### Ethics statement

The study was reviewed and granted approval (#H-43232) by the Institutional Review Board (IRB) Staff at Boston University who determined that the study qualifies for an exemption under the policies and procedures of the Human Research Subjects Program, category 2. See https://www.bumc.bu.edu/ohra/hrpp-policies/hrpp-policies-procedures/#10.2.4. All experiments were performed in accordance with these guidelines. We obtained written consent (via a closed-form survey question) from respondents prior to their beginning the survey.

The preregistration for the study’s experimental design and empirical expectations is available at https://osf.io/kx43r.

### Data

Data for this study come from a YouGov-fielded survey of N = 2,200 US adults (18 and older). YouGov used propensity score matching procedures to ensure that our sample is nationally representative. The firm did this by first taking a random sample of respondents from the nationally representative American Community Survey (ACS), then they used propensity score matching techniques to determine which members of its large online opt-in panel most closely resemble each of the cases drawn from the ACS and invited those individuals to participate in the study.

YouGov also provided us with post-stratification weights that account for any remaining deviations between the survey sample and demographic benchmarks from the US Census. We apply these weights when conducting our multivariate analyses. According to an independent analysis by the Pew Research Center, YouGov has outperformed other online data vendors on accuracy [29].

### Design

We tested our hypotheses by embedding a three-armed randomized controlled trial (RCT), into a nationally representative public opinion survey. In it, we exposed respondents to one of two co-constitutive risk (CCR) messages (vs. a pure control message pertaining to the history of baseball). A limitation of this design was that we did not include a condition with a typical health communication message emphasizing a single risk to compare to the co-constitutive risk conditions. This can be addressed in future research.

**Fig 1:**
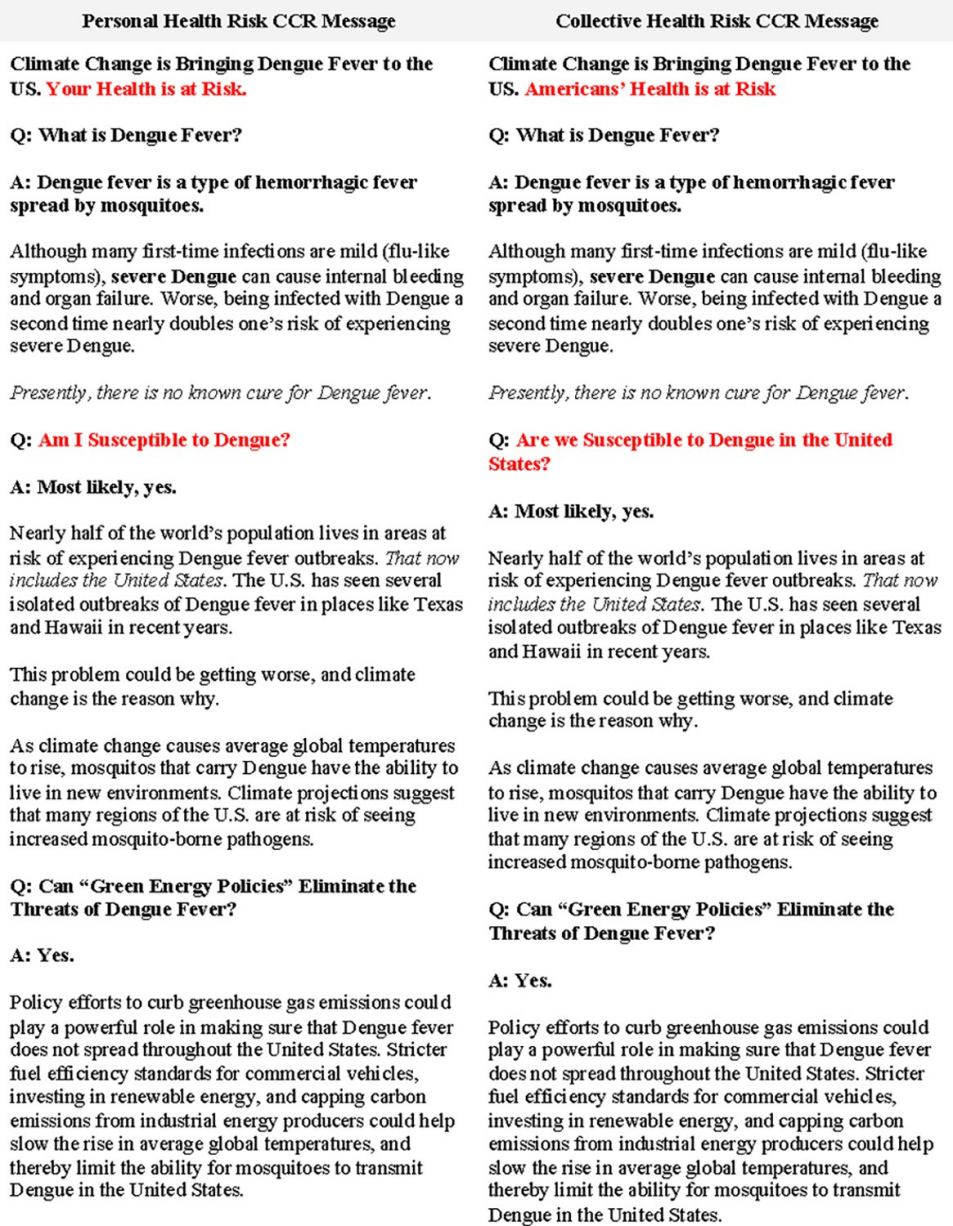
Experimental Treatment Materials. Participants who were randomly assigned to the personal risk condition saw the message on the left, and participants who were randomly assigned to the collective risk condition saw the message on the right.

Both CCR messages emphasized the risk of contracting dengue and how that risk will increase given global climate change. However, the messages varied in cultural cognitive framing, such that one CCR message (“individual risk”) emphasizes the spread of mosquito-borne infection as a risk to one’s *personal* health, while another (“collective risk”) emphasizes the *public* health risks of mosquito-borne infection. Fig 1 provides a side-by-side comparison of each CCR message, with differences in cultural cognitive framing highlighted in red.

### Analytic strategy

We test Hypotheses 1 and 2 by constructing a series of ordered logistic regression models that regress ordinal indicators of each outcome described in the hypotheses on dichotomous indicators of whether respondents were assigned to either of the two messages presented in Fig 1 (with assignment to the pure control group serving as an analytical reference category). If our theoretical expectations are supported, both of these indicators should be positively (i.e., β > 0) and significantly (at the *p* < 0.05 level, two-tailed) associated with each of the aforementioned outcome variables. Note that, due to an oversight in our original pre-analysis plan, we code support for dengue vaccine mandates as a dichotomous outcome variable and model these views via logistic regression. We do this both due to the lack of a clear causal ordering between the variable’s penultimate categories as well as our substantive interest in support for vaccine mandates (the first response option).

Note that, because we randomly assign respondents into each treatment and the control, we do not expect to observe differences in demographic makeup across groups. Indeed, in a series of randomization checks included in the Supporting Information (S1 Table)—calculated on the basis of respondents’ gender identity, racial identity, age, educational attainment, and party identification—we find no evidence some respondents were more likely than others to be assigned into either of our two treatment groups (vs the control) than others. Correspondingly, we do not condition on any covariates in our models.

We test Hypothesis 3 by amending the ordered logistic regression models described above to *interact* each treatment assignment indicator with a multi-item index denoting the extent to which people hold individualistic or communitarian cultural cognitive worldviews.

If our theoretical expectations are supported, we would expect that the interaction term denoting that more individualistic people who were assigned to the “personal risk” condition will be (a) positively and significantly associated with increases in each of the outcome variables listed in Table 1, and (b) that these effects will be significantly larger than those for the more individualistic people who were assigned to the “communal risk” condition, as well as for all communitarians assigned to the “personal risk” condition. We expect to observe a complimentary pattern for communitarians assigned to the “communal risk” condition.

**Table 1:** Vaccination-related Outcomes. Descriptive statistics (weighted proportions from the study’s control group) are presented in brackets next to each response option. Response option order was rotated between participants. For Dengue Fever Vaccine Mandate options, only A-C were rotated.

Outcome	Question Text	Response Options
Dengue Fever Vaccine Mandate	Some argue that the US should require vaccination for *all children living in the United States* who have previously been infected with dengue. Others agree that dengue fever vaccination in the US should be expanded to all children who have previously been infected, but think that the decision to vaccinate should be a matter of personal choice. Still others argue that vaccination eligibility should not be expanded at all. What about you? Do you think that eligibility for dengue fever vaccination…	<A> Should be both expanded and mandatory for all US children aged 9–16 who have previously been infected with dengue fever. [24%] <B> Should be expanded to all US children aged 9–16 who have been previously infected with dengue fever, but optional [37%] <C> Should not be expanded at all [18%] <D> Not sure [21%]
Prospective Vaccine Uptake	If federal health agencies were to deem a new dengue fever vaccine safe and effective for adults (aged 16 or older) how likely would you be to receive the vaccine?	<1> Very likely [24%] <2> Somewhat likely [26%] <3> Not too likely [28%] <4> Not likely at all [21%]
Vaccine Research & Development	Please tell us the extent to which you agree or disagree with the following statement: Even if it means raising taxes, the federal government invest in research to develop vaccines for mosquito borne illnesses (e.g., dengue fever, Zika).	<1> Strongly agree [10%] <2> Agree [18%] <3> Neither agree nor disagree [27%] <4> Disagree [29%] <5> Strongly disagree [15%]

### Measures

#### Outcome variables

There are two primary sets of outcomes in this study. The first set are a series of ordinal indicators of the extent to which survey respondents support policies aimed at promoting vaccination against neglected tropical diseases like dengue fever, and we refer to these as the immediate outcomes (Hypothesis 1). These policies include support for research and development into vaccination for mosquito borne illnesses, the expansion of dengue vaccine requirements for children (as dengue fever vaccination is currently only approved for children aged 9–16) and (adult) respondents’ hypothetical intentions to vaccinate against dengue, should a vaccine for adults be approved by federal regulators. Each vaccine-related question was preceded by the following preamble:

*Aside from taking care to avoid mosquito bites, one simple way to prevent getting sick with dengue is to get vaccinated against the disease. Safe and effective vaccines are available for children aged 9–16, who have previously been infected with dengue. At this time only those traveling to areas where dengue is common are eligible for vaccination. As rising global temperatures attributable to climate change may increase the likelihood that mosquito borne illness become more common in the United States, some argue that federal regulators ought to rethink this policy*.

The question wording and response options are available in Table 1. Note that the first outcome variable, Dengue Fever Vaccine Mandate, was coded such that support for mandate expansion (option A) takes on a value of 1, with all other variables taking on a value of 0.

The second set of outcome variables are ordinal indicators measuring the extent to which respondents’ support policies aimed at mitigating the effects of climate change and we refer to these as the superordinate outcomes (Hypothesis 2). These policies (adapted from [30]) include support for research into sources of renewable energy, carbon dioxide regulation, carbon dioxide emission limits on power plants, and investment in diversifying the sources of energy production. Full question wording and response option information is available in Table 2.

**Table 2:** Climate-related Outcomes. Descriptive statistics (weighted proportions from the study’s control group) are presented in brackets next to each response option. Response option order was rotated between participants.

Outcome	Question Text	Response Options
Renewable Energy Research & Development	To what degree, if at all, do you think that the US federal government should fund research into renewable energy sources, such as solar and wind power?	<1> Strongly support [8%] <2> Support [9%] <3> Neither supp. nor opp. [20%] <4> Oppose [27%] <5> Strongly oppose [35%]
Greenhouse Gas (CO2) Regulation	To what degree, if at all, do you think that the US federal government should regulate carbon dioxide (the primary greenhouse gas) as a pollutant?	<1> Strongly support [9%] <2> Support [9%] <3> Neither supp. nor opp. [24%] <4> Oppose [27%] <5> Strongly oppose [30%]
Power Plant (Coal) Emission Limits	To what degree, if at all, do you think that the US federal government should set strict carbon dioxide emission limits on coal-fired power plants, even if it caused the cost of electricity to increase for companies and consumers?	<1> Strongly support [12%] <2> Support [17%] <3> Neither supp. nor opp. [24%] <4> Oppose [25%] <5> Strongly oppose [23%]
Energy Source Diversification	To what degree, if at all, do you think that the US federal government should require utility companies to produce at least 20% of their electricity from wind, solar, or other renewable energy sources, even if it costs the average household an additional $100 per year?	<1> Strongly support [15%] <2> Support [17%] <3> Neither supp. nor opp. [21%] <4> Oppose [25%] <5> Strongly oppose [22%]

Note that all variables were scored such that the maximum ordinal category on each one corresponds to the highest possible levels of support for both the immediate (vaccine) and superordinate (climate) outcomes.

#### Independent variables

The primary explanatory variables used to test Hypotheses 1 and 2 are dichotomous indicators of whether survey respondents were assigned to read one of the two experimental treatments versus the control group.

When testing Hypothesis 3, we add an intervalized, multi-item index assessing respondents’ placement on an individualist-communitarian continuum (M = 0.56, SD = 0.20) [23]. We construct this index by averaging responses across a series of 18 items—derived from Kahan et al. [23,26,31]—that ask respondents to assess whether they agree or disagree with a series of statements designed to measure individualistic or communitarian cultural worldviews (e.g., views that the government interferes “far too much in our everyday lives”).

We score the resulting (untransformed) scale to range from 0–1, such that a score of 1 is indicative of expressing strong individualistic preferences (ɑ = 0.93). For simplicity, we will refer to this measure as *individualism* throughout our analyses. A full list of items used to build this scale can be found in the attached supplement (S1 Appendix).

## Results

### Results of pre-registered analyses

We begin by assessing the degree to which exposure to CCR messaging is associated with increased support for pharmaceutical interventions against the health risks posed by dengue fever (Immediate Outcomes; Hypothesis 1), as well as support for climate change mitigation policies (Superordinate Outcomes; Hypothesis 2).

The results of the multivariate models devised to test these hypotheses (see Analytical Strategy) are presented in Table 3. Note that although we did not state *a priori* that we expected to observe asymmetries in effectiveness across differences in cultural cognitive message framing, we nevertheless account for this possibility in our models (as identified in our pre-analysis plan).

**Table 3:** Pre-Registered Experimental Treatment Effect Analyses (Main Effects).

	Immediate Outcomes (Vaccine Attitudes: H1)			Superordinate Outcomes (Climate Policy Attitudes: H2)			
	Vax Mandates	Vax Uptake	Vax R&D	Renew	CO2	Coal	Diverse
Exp: Personal Risk	-0.11	0.06	-0.03	-0.06	-0.08	-0.09	-0.19
	(0.15)	(0.11)	(0.11)	(0.11)	(0.11)	(0.11)	(0.10)
Exp: Collective Risk	0.06	0.25*	0.01	0.05	-0.03	0.09	-0.02
	(0.15)	(0.12)	(0.11)	(0.11)	(0.11)	(0.11)	(0.11)
τ_1_	1.15*	-1.10*	-1.97*	-2.32*	-2.26*	-1.83*	-1.63*
	(0.11)	(0.09)	(0.09)	(0.11)	(0.10)	(0.09)	(0.09)
τ_2_	-	-0.02	-0.92*	-1.54*	-1.48*	-0.93*	-0.79*
		(0.08)	(0.08)	(0.09)	(0.09)	(0.08)	(0.08)
τ_3_	-	1.33*	0.16*	-0.51*	-0.29*	0.05	0.16*
		(0.09)	(0.08)	(0.08)	(0.08)	(0.08)	(0.08)
τ_4_	-	-	1.71*	0.61*	0.87*	1.19*	1.24*
			(0.09)	(0.08)	(0.08)	(0.08)	(0.08)
N	2200	2199	2200	2200	2200	2200	2200

Note. Logistic (Column 2) and ordered logistic (Columns 3–8) regression parameters presented, with standard errors in parentheses. Survey weights applied.

The results presented in Table 3 provide mixed support for Hypothesis 1. Consistent with Hypothesis 1, we find that participants who were exposed to CCR messages that emphasize the communal health risks of dengue fever (vs. exposure to the pure control) had greater prospective vaccination intentions (β = 0.25, *p* = 0.03; Column 2). Transforming that parameter estimate into predicted probabilities that hold all other covariates at their sample means, we find that this corresponds to approximately a 4-percentage point increase in the probability that respondents say that they are “very likely” to vaccinate across the treatment (25%) and control (21%) conditions. We also note that the results are correctly signed, albeit non-significant, with respect to support for policies aimed at expanding dengue vaccine requirements (Column 1) as well as investment in dengue vaccine research (Column 3). In contrast, although we made no *a priori* predictions about differences between message frames, we find that personal risk CCR message bore no significant associations with any of the outcome variables used to test Hypothesis 1.

Furthermore, and surprisingly, we find no evidence in support of Hypothesis 2 (Columns 4–7). Neither personal nor collective risk messages were significantly associated with increased support for policies aimed at mitigating climate change.

Next, we consider the possibility that the effectiveness of CCR messaging might be conditional on respondents’ cultural cognitive worldviews; such that individualists are more responsive to personal risk messages and collectivists are more responsive to communal risk messages (Hypotheses 3a and 3b). We present the aforementioned interactive models (see Analytical Strategy) in Table 4.

**Table 4:** Pre-Registered Experimental Treatment Effect Analyses (Moderated Effects).

	Immediate Outcomes (Vaccine Attitudes: H1)			Superordinate Outcomes (Climate Policy Attitudes: H2)			
	Vax Mandates	Vax Uptake	Vax R&D	Renew	CO2	Coal	Diverse
Exp: Personal Risk	0.22	0.54	0.54	0.06	-0.13	0.23	-0.19
	(0.41)	(0.34)	(0.32)	(0.39)	(0.38)	(0.37)	(0.37)
Exp: Collective Risk	0.11	0.82*	0.93*	0.05	0.19	0.48	0.50
	(0.41)	(0.35)	(0.33)	(0.40)	(0.39)	(0.37)	(0.39)
Individualism	-4.20*	-5.13*	-5.58*	-8.11*	-8.43*	-8.16*	-7.78*
	(0.57)	(0.41)	(0.40)	(0.49)	(0.51)	(0.46)	(0.49)
Personal X Ind.	-0.62	-0.74	-0.92	-0.18	0.12	-0.60	-0.10
	(0.80)	(0.56)	(0.53)	(0.61)	(0.60)	(0.61)	(0.62)
Collective X Ind.	-0.01	-0.87	-1.59*	0.04	-0.38	-0.61	-0.95
	(0.78)	(0.58)	(0.56)	(0.64)	(0.63)	(0.61)	(0.65)
τ_1_	-1.03*	-4.21*	-5.64*	-7.81*	-7.98*	-7.26*	-6.69*
	(0.29)	(0.26)	(0.27)	(0.36)	(0.37)	(0.32)	(0.33)
τ_2_	-	-2.84*	-4.27*	-6.74*	-6.84*	-5.86*	-5.42*
		(0.25)	(0.25)	(0.34)	(0.34)	(0.30)	(0.31)
τ_3_	-	-1.20*	-2.84*	-5.22*	-5.05*	-4.40*	-4.05*
		(0.24)	(0.24)	(0.31)	(0.32)	(0.28)	(0.29)
τ_4_	-	-	-0.93*	-3.64*	-3.42*	-2.83*	-2.62*
			(0.23)	(0.29)	(0.30)	(0.27)	(0.28)
N	2200	2199	2200	2200	2200	2200	2200

Note

*p < 0.05; two-tailed; Logistic (Column 2) and ordered logistic (Columns 3–8) regression parameters presented, with standard errors in parentheses. Survey weights applied.

Table 4 again reveals mixed evidence in favor of the conditional effectiveness of CCR messaging with respect to its effects on vaccine attitudes. In this case, collectivists exposed to public health-focused CCR messages were significantly more likely to favor investing in the development of dengue fever vaccines (β = 1.59, *p* = 0.01). Substantively, these effects correspond to an approximately 15-percentage point decrease in the predicted probability that respondents who express the strongest observed levels of collectivism (i.e., a score of 0 on the individualism scale) indicate that they “strongly agree” with raising taxes to fund dengue vaccine research across the treatment (87%) versus control groups (72%).

However, we detect no evidence of moderation by cultural cognitive worldviews on vaccination intentions or support for expanding vaccine requirements. Likewise, as was the case when testing Hypothesis 2, we find no evidence of effects of CCR messaging across cultural cognitive worldviews on support for climate change mitigation policy. Notably, however, individualism scores negatively predicted vaccine and climate policy attitudes, across the message conditions.

### Post Hoc (Exploratory) analyses

To this point, our pre-registered analysis plan has yielded mixed results in testing our theoretical expectations regarding the efficacy of CCR messaging. We recognize, however, that we may have overlooked a potential moderating influence on CCR messaging effectiveness when registering our theoretical expectations.

Specifically, it may be the case that CCR treatments inspire support for both pharmaceutical interventions and climate change mitigation policy when we consider individuals’ beliefs about anthropogenic climate change. People who accept the scientific consensus that changes in the planet’s climate are caused by human activities (anthropogenic climate change acceptance; ACC) tend to be more likely to express concern about its harmful effects on human life and support policies to curb greenhouse gas emissions [32].

Correspondingly, individuals who accept and are already concerned about ACC may exhibit a comparatively lower capacity to be influenced by our CCR messages (i.e., a “ceiling effect”) because they already express a strong desire to take action to lessen its effects. We therefore hypothesize that CCR message exposure effectiveness may be further moderated by ACC acceptance, such that only those who doubt the reality of human-caused climate change could be influenced by the messages.

**Fig 2:**
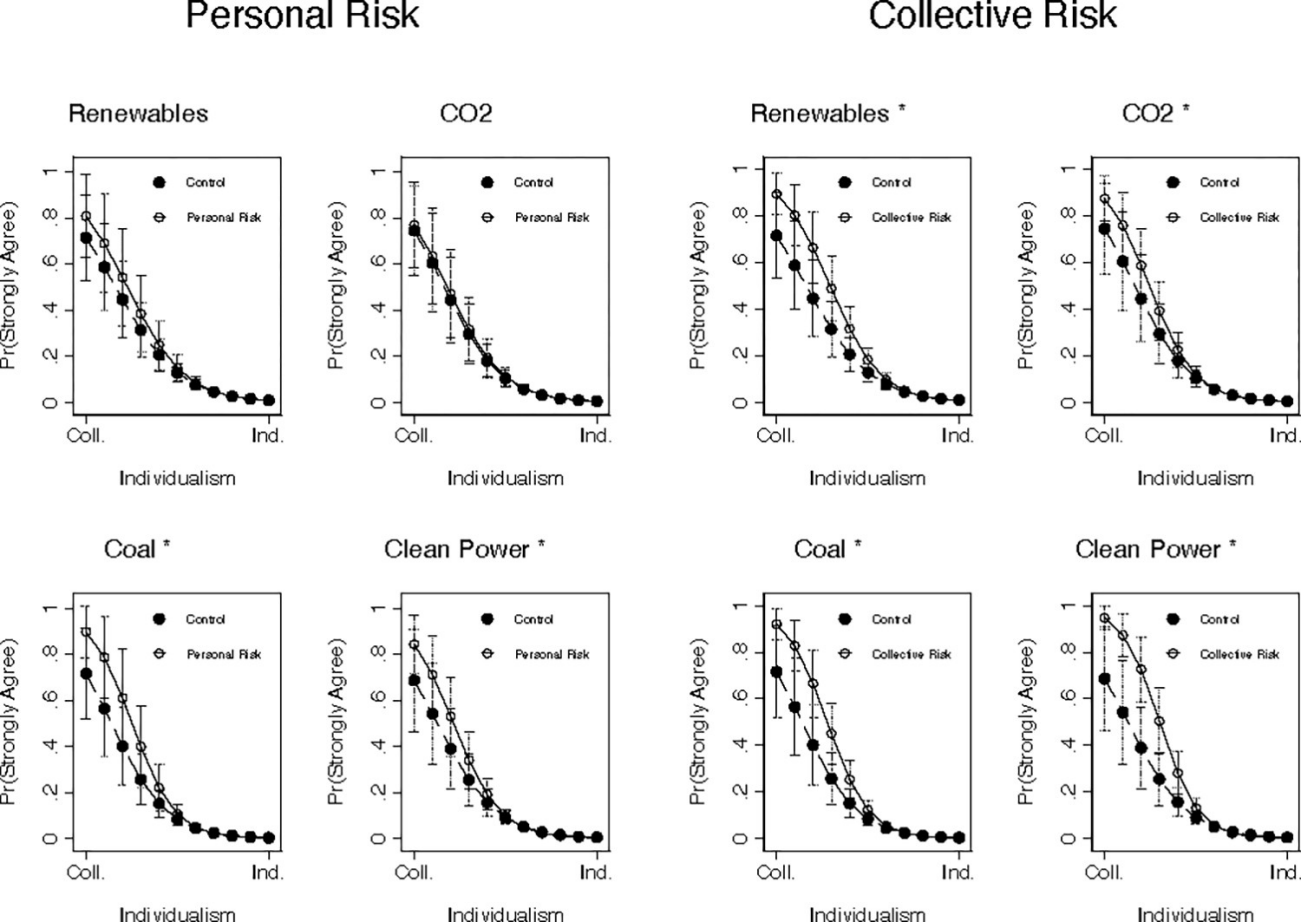
The Moderated Effects of CCR Message Exposure on Climate Policy Attitudes Among ACC Skeptics. Predicted probabilities (circles) presented with 95% confidence intervals extending out from each one. Predictions are calculated based on the models presented in S3 Table, and hold all covariates at their sample means.

We test this possibility by amending the models presented in Tables 3 and 4 to interact each treatment indicator (the models in Table 3), as well as the treatment by cultural worldview interactions (the models in Table 4), with a dichotomous indicator of whether or not survey respondents believe that “the earth is getting warmer mostly because of human activity such as burning fossil fuels” (see the S1 Appendix for complete question-wording information). The results are presented in S2 and S3 Tables and summarized graphically in Fig 2.

*Post hoc*, we expected that CCR messages would most effectively influence the attitudes and behaviors of those who do not already view climate change as human-caused. On the contrary, we find no evidence that exposure to either of the two CCR message treatments is associated with increased support for pharmaceutical interventions to combat dengue fever or climate change mitigation policy (in all cases, *p* > 0.05; two-tailed). However, consistent with Hypothesis 3, we find that ACC beliefs moderate message effectiveness, especially concerning the outcomes that measure the treatments’ *influence* on climate attitudes (as a reminder, please see Tables 1 and 2 in the method section for complete question-wording information).

Specifically, we report in the S3 Table that the three-way interaction between exposure to personal risk CCR messages, ACC acceptance, and individualistic attitudes is associated with increased support for additional government regulation of coal-fired power plants (β = 3.81, *p* = 0.02) and diversifying utilities’ clean power sources (β = 4.85, *p* < 0.01).

We find an analogous pattern of results across all four climate policy outcomes for the CCR messages that emphasize collective risk. For those messages, CCR exposure is associated with significantly greater levels of support for government investment in renewable energy (β = 3.69, *p* = 0.02), as well as increased government regulation on coal-fired power plants (β = 3.59, *p* = 0.02) and clean power diversification (β = 4.68, *p* < 0.01). Exposure to the collective risk messages is also associated with support for government regulation of CO2 emissions (β = 2.93), although this effect only approaches conventional levels of two-tailed significance (*p* = 0.07). Treatment exposure, however, has no statistically discernible impact on support for pharmaceutical interventions when accounting for the possibility of moderation by ACC beliefs (*p* > 0.10 in all cases).

Of course, these three-way interactive terms are difficult to interpret on their own. Correspondingly, Fig 2 plots the predicted probability (y-axis) of indicating strong levels of support for each of the aforementioned climate policies for individuals who were exposed to each CCR message (solid vs. dashed lines, with personal risk messaging presented on the left-hand side of the figure, and collective risk messaging presented on the right), across levels of individualistic attitude endorsement (x-axis), for those who express skepticism about ACC.

Note that we display predictions from all of the aforementioned models for ease of visual comparison. However, following Brambor and colleagues [33], we strongly caution against interpreting substantive effects from models that produced non-significant interaction terms. Thus, for reference, we suffix all significant interactions with an asterisk.

Fig 2 demonstrates, somewhat surprisingly, that exposure to our CCR treatments—irrespective of cultural cognitive framing—is associated with significantly more robust support for pro-climate policies for individuals who hold more collectivist worldviews (as demonstrated by both the elevated position of the dashed line, as well as the non-overlapping confidence intervals). For example, the predicted probability of strongly supporting clean power regulations is 90% for people who express ACC skepticism, hold strongly collectivistic worldviews, and were exposed to the collective risk CCR message, compared to 71% in the control group (a 19-percentage point increase). As Fig 2 shows (contrary to our *post hoc* expectations), we observe an analogous pattern for messages emphasizing personal risk.

While these results comport with our theoretical expectations regarding the asymmetric appeal of collective risk messages to those with less individualistic worldviews, we were surprised that our personal risk messages did not produce an analogous effect pattern among those with more individualistic worldviews. It seems likely that, because all of the policies under investigation require some level of government action, which could be seen as infringing on individual freedoms, only those who value collective approaches to solving health and climate issues are responsive to our treatments. Although we did not specify this mechanism *a priori*, this could be a fascinating area for future investigation.

## Discussion

Our results document the effectiveness of co-constitutive risk messaging (CCR), a method of *One Health Communication*, on taking both immediate action (i.e., on pro-vaccine policy and uptake intentions) and supporting superordinate action (i.e., on climate change mitigation policy) to stop the spread of climate-facilitated infectious disease. These effects occur through a variety of different channels. CCR message exposure exhibits both main and moderated (by cultural cognitive orientation) influence on the immediate outcomes of pro-vaccine attitudes and behavior. Still, it appears to have no effects on the more superordinate outcomes of climate policy attitudes. However, when we account for the possibility of “ceiling effects” among those who accept anthropogenic climate change (ACC) as real, we document strong effects of exposure to CCR messaging among those who hold collectivist cultural worldviews.

Surprisingly, and inconsistent with our pre-registered theoretical expectations, we find little evidence that those with more individualistic worldviews were more responsive to CCR threats that emphasized the personal health risks of climate change. Although we hesitate to speculate post hoc as to why our theoretical expectations were not borne out in the data, one possibility could be, as we suggested in the results section, that the proposed government interventions, which can be seen as infringing on personal freedoms, were more salient to individualists than the risks posed by dengue from anthropogenic climate change. Relatedly, it is also possible that our treatments did not emphasize clearly enough either the severity or (increasing) probability of getting sick with insect-borne illnesses. We see this as both a limitation of our research and an opportunity for future work to develop, pilot, and test different messages emphasizing the personal health risks of climate change.

More generally, we tend to observe the strongest effects of CCR messaging among those less convinced that climate change results from human activities. As noted throughout the paper, we suspect that moderation by ACC beliefs reflects the idea that those who already accept climate change as real and human-caused may not have much opportunity to update their health and/or climate-related beliefs in response to CCR messaging, i.e., because they are already precisely the types of people who report that they favor taking action to lessen the health risks borne by climate change. We, therefore, encourage future research in this area to anticipate the possibility of ACC belief moderation when devising extensions of this messaging approach and to always measure attitudes about the causes of climate change when researching climate messaging.

Taken together, these findings offer admittedly mixed support for our pre-registered hypotheses. However, we believe that they represent an important first step in assessing the viability of CCR messaging as a One Health Communication approach.

Growing scientific evidence highlighting the negative impacts of climate change [34] has not convinced many US Americans of the urgency of climate action [35]. Past research indicates that a non-trivial proportion of US Americans are either concerned about climate change (i.e., believe that the climate is changing but tend to believe that climate impacts are still distant in time and space), cautious about climate change (have not yet made up their minds); disengaged from climate change (know little about it); and doubtful about climate change (they do not think global warming is happening or they believe it is just a natural cycle) [36]. That is despite scholars making increasing connections between climate change and public health in recent years [37,38]. Sadly, lack of policy action on issues with a long-time horizon is frequently a feature of democratic politics, as citizens are often strongly biased toward policies addressing present problems over long-term ones [39].

We believe that in this context, CCR messaging and the broader One Health Communication paradigm offer some hope by linking climate change to a specific disease, which can help to make the non-obvious effects of climate change real and immediate in the mind of many Americans, especially those who are less committed to climate action due to their skepticism about global warming. Although more research is needed, CCR messaging could potentially even influence the citizens who are already alarmed about climate change by motivating them towards more concrete climate action. This paradigm enables a more comprehensive and potentially effective engagement with diverse audiences on complex, interlinked issues beyond climate change.

Of course, these are results from a single study offering only one CCR messaging application. Moreover, our work is presented in a single national context (the United States). We urge strategic communication researchers in both the US and beyond to consider whether these strategies hold promise in other national and cross-cultural contexts.

Beyond considering cross-national applications of CCR messaging, future work should expand on our approach in other One Health-related applications. Future work might also consider administering a “stronger” experimental treatment, i.e., because although we detect important experimental treatment effects, our manipulation featured only a few minor word changes in the experimental primes. More robust treatment vignettes would more fully reveal the effects that CCR messaging might have. Finally, future efforts to apply CCR messaging should account for different moderators, including accounting for the possibility that some worldviews and psychological predispositions might make someone more persuadable than others [40], collectivists could be more persuadable than individualists simply because they tend to be higher on openness, an important psychological factor determining persuadability.

Still, the results presented in this manuscript hold promise for strategic health and environmental communication. Principally, our work in what we term *One Health Communication* emphasizes that–in some respects–health and environmental communication represent two sides of the same proverbial coin. In other words, because climate risks can beget concerns about the spread of infectious disease, we encourage strategic communicators to make an effort to identify areas in which these two concerns ought to be raised in concert with one another.

To do this, one concrete step that strategic health and climate communicators can take in the short term is to conduct pilot survey-based RCTs that assess the efficacy of CCR messages that fuse climate and health risks into the same strategic messages. In addition to expanding on the scope of health risks assessed in this piece (e.g., the spread of other insect-borne diseases attributable to climate change, such as Lyme Disease), this applied work might also consider assessing the efficacy of content elements not tested in the present research; e.g., the possibility that providing visual risk-related imagery–like pictures of insect vectors and/or infected humans–may evoke comparatively stronger feelings of anxiety in those who view them than those viewing messages lacking that imagery.

After determining which content elements might enhance the effectiveness of CCR messages in an expanded range of public health domains, communicators might then consider “scaling up” pilot messaging approaches into larger (and more costly) field experimental interventions conducted on web, print, televised, and/or socially mediated platforms. This would allow communicators to assess the external generalizability of the effectiveness of CCR messaging. This would also enable communicators to consider how different content elements might enhance CCR messages’ abilities to both induce feelings of risk and, correspondingly, inspire behavioral action to mitigate those risks.

Overall, we see our work not as the “final word” on CCR messaging but as offering a blueprint for future research in this area. We look forward to future efforts to build on the analyses offered here to better understand the viability of this and other One Health Communication strategies.

## Supporting information

S1 Table Supplemental Randomization Checks. (DOCX)

S2 Table Supplemental Analyses. Post Hoc Reestimation of Table 3, with Moderation by ACC beliefs.
(DOCX)

S3 Table Supplementary Analyses. Post Hoc Reestimation of Table 4, with Moderation by ACC beliefs.
(DOCX)

S1 Appendix Item wording used in the survey. (DOCX)
