# Supplementary material for: Combating climate-induced health threats through Co-Constitutive Risk (CCR) Messaging: A One Health Communication approach
Source: PLoS Negl Trop Dis. 2024 Dec 2;18(12):e0012676. doi: 10.1371/journal.pntd.0012676 (PMC11637427; doi:10.1371/journal.pntd.0012676)
Supplement: S1 Table — (DOCX) [file pntd.0012676.s001.docx]

# S1 Table. Supplemental Randomization Checks.

|  | **Treatment** | **Personal** | **Collective** |
| --- | --- | --- | --- |
| Gender ID = Woman | 0.54  [0.50, 0.58] | 0.54  [0.50, 0.58] | 0.56  [0.52, 0.60] |
| Racial ID = Black | 0.12  [0.10, 0.15] | 0.12  [0.10, 0.15] | 0.12  [0.09, 0.14] |
| Ethnicity ID = Hispanic | 0.10  [0.08, 0.12] | 0.11  [0.09. 0.14] | 0.09  [0.07, 0.12] |
| College Educated | 0.42  [0.39, 0.46] | 0.40  [0.36, 0.44] | 0.39  [0.35, 0.42] |
| PID = Democrat | 0.44  [0.40, 0.47] | 0.42  [0.38, 0.45] | 0.45  [0.41, 0.48] |
| PID = Republican | 0.38  [0.34, 0.42] | 0.38  [0.34, 0.41] | 0.34  [0.31, 0.38] |
| Mean Age (in years) | 51  [50, 52] | 51  [49, 52] | 51  [49, 52] |

*Note.* Unweighted sample proportions (rows 1-6) and means (row 7) across experimental conditions presented, with 95% confidence intervals in brackets. Non-significance (demonstrative of successful randomization) is designated by overlapping 95% confidence intervals across the values presented in each row.
