# Supplementary material for: Combating climate-induced health threats through Co-Constitutive Risk (CCR) Messaging: A One Health Communication approach
Source: PLoS Negl Trop Dis. 2024 Dec 2;18(12):e0012676. doi: 10.1371/journal.pntd.0012676 (PMC11637427; doi:10.1371/journal.pntd.0012676)
Supplement: S2 Table — Post Hoc Reestimation of Table 3, with Moderation by ACC beliefs. (DOCX) [file pntd.0012676.s002.docx]

# S2 Table. Supplemental Analyses. Post Hoc Reestimation of Table 3, with moderation by ACC beliefs.

##

|  | **Immediate Outcomes**  **(Vaccine Attitudes: H1)** | | | **Superordinate Outcomes**  **(Climate Policy Attitudes: H2)** | | | |
| --- | --- | --- | --- | --- | --- | --- | --- |
|  | Vax. Mandates | Vax. Uptake | Vax. R&D | Renew. | CO2 | Coal | Diverse. |
| Exp: Personal Risk | -0.25 | 0.04 | -0.12 | -0.03 | 0.01 | -0.12 | -0.18 |
|  | (0.29) | (0.16) | (0.15) | (0.15) | (0.15) | (0.16) | (0.15) |
| Exp: Collective Risk | 0.07 | 0.27 | 0.04 | 0.14 | -0.06 | 0.09 | -0.03 |
|  | (0.27) | (0.17) | (0.15) | (0.15) | (0.15) | (0.15) | (0.16) |
| Accept ACC | 1.37* | 1.79* | 2.08* | 3.14* | 2.98* | 2.68* | 2.54* |
|  | (0.24) | (0.17) | (0.16) | (0.18) | (0.18) | (0.16) | (0.16) |
| Personal X ACC | 0.27 | 0.17 | 0.30 | -0.01 | -0.05 | 0.08 | 0.01 |
|  | (0.34) | (0.23) | (0.21) | (0.24) | (0.23) | (0.24) | (0.22) |
| Collective X ACC | -0.01 | 0.03 | -0.01 | -0.25 | 0.04 | -0.01 | -0.06 |
|  | (0.33) | (0.24) | (0.23) | (0.23) | (0.23) | (0.22) | (0.23) |
|  |  |  |  |  |  |  |  |
| τ_1_ | 1.92* | -0.44* | -1.38* | -1.61* | -1.56* | -1.14* | -0.91* |
|  | (0.20) | (0.12) | (0.11) | (0.11) | (0.12) | (0.11) | (0.11) |
| τ_2_ | - | 0.84* | -0.18 | -0.73* | -0.67* | -0.04 | 0.12 |
|  |  | (0.12) | (0.10) | (0.10) | (0.11) | (0.11) | (0.11) |
| τ_3_ | - | 2.45* | 1.20* | 0.65* | 0.97* | 1.35* | 1.44* |
|  |  | (0.13) | (0.11) | (0.10) | (0.12) | (0.11) | (0.11) |
| τ_4_ | - | - | 3.11* | 2.48* | 2.78* | 2.93* | 2.87* |
|  |  |  | (0.13) | (0.13) | (0.14) | (0.13) | (0.12) |
| N | 2200 | 2199 | 2200 | 2200 | 2200 | 2200 | 2200 |

** p < 0.05; two-tailed*

*Note. Logistic (Column 2) and ordered logistic (Columns 3-8) regression parameters presented, with standard errors in parentheses. Survey weights applied.*
