# Supplementary material for: Combating climate-induced health threats through Co-Constitutive Risk (CCR) Messaging: A One Health Communication approach
Source: PLoS Negl Trop Dis. 2024 Dec 2;18(12):e0012676. doi: 10.1371/journal.pntd.0012676 (PMC11637427; doi:10.1371/journal.pntd.0012676)
Supplement: S1 Appendix — (DOCX) [file pntd.0012676.s004.docx]

# S2 Appendix. Item wording used in the survey.

## *ACC Beliefs*

Which of these three statements about the Earth’s temperature comes closest to your view?

<1> The Earth is getting warmer mostly because of human activity such as burning fossil fuels

<2> The Earth is getting warmer mostly because of natural patterns in the Earth’s environment

<3> There is no solid evidence that the Earth is getting warmer

<4> Don’t know

## *Individualism Index*

Preamble. People in our society often disagree about how far to let individuals go in making decisions for themselves. How strongly do you agree or disagree with each of these statements?

[CODING NOTE: REVERSE CODE “C” ITEMS; C = COLLECTIVIST; I = INDIVIDUALIST]

*1.* *IINTRSTS. The government interferes far too much in our everyday lives.*

*2.* *CHARM. Sometimes government needs to make laws that keep people from hurting themselves.*

*3.* *IPROTECT. It's not the government's business to try to protect people from themselves.*

*4.* *IPRIVACY. The government should stop telling people how to live their lives.*

*5.* *CPROTECT. The government should do more to advance society's goals, even if that means limiting the freedom and choices of individuals.*

*6.* *CLIMCHOI. Government should put limits on the choices individuals can make so they don't get in the way of what's good for society.*

7. INEEDS. Too many people today expect society to do things for them that they should be doing for themselves.

8. CNEEDS. It's society's responsibility to make sure everyone's basic needs are met.

9. INEEDY. It's a mistake to ask society to help every person in need.

10. CRELY. People should be able to rely on the government for help when they need it.

11. IRESPON. Society works best when it lets individuals take responsibility for their own lives without telling them what to do.

12. ITRIES. Our government tries to do too many things for too many people. We should just let people take care of themselves.

13. IFIX. If the government spent less time trying to fix everyone's problems, we'd all be a lot better off.

14. IENJOY. People who are successful in business have a right to enjoy their wealth as they see fit.

15. IMKT. Free markets--not government programs--are the best way to supply people with the things they need.

16. IPROFIT. Private profit is the main motive for hard work.

17. IGOVWAST. Government regulations are almost always a waste of everyone's time and money.

<1> Strongly Agree

<2> Agree

<3> Neither Agree nor Disagree

<4> Disagree

<5> Strongly Disagree
